# Evolutionary adaptation to aquatic lifestyle in extinct sloths can lead to systemic alteration of bone structure

**DOI:** 10.1098/rspb.2018.0270

**Published:** 2018-05-09

**Authors:** Eli Amson, Guillaume Billet, Christian de Muizon

**Affiliations:** 1 Museum für Naturkunde, Leibniz-Institut für Evolutions- und Biodiversitätsforschung, Invalidenstraße 43, Berlin 10115, Germany; 2 AG Morphologie und Formengeschichte, Institut für Biologie, Humboldt Universität zu Berlin, Philippstraße 13, Berlin 10115, Germany; 3 Bild Wissen Gestaltung, Ein Interdisziplinäres Labor, Humboldt Universität zu Berlin, Sophienstraße 22a, Berlin 10178, Germany; 4 Département Origines et Évolution, Muséum national d'Histoire naturelle, Centre de Recherche sur la Paléobiodiversité et les Paléoenvironnements-CR2P (UMR 7207, CNRS, MNHN, UPMC, Sorbonne Université), 8 rue Buffon, Paris 75005, France

**Keywords:** bone mass increase, evolutionary adaptation, phenotypic plasticity, physiological adjustment, *Thalassocnus*, turbinates

## Abstract

Through phenotypic plasticity, bones can change in structure and morphology, in response to physiological and biomechanical influences over the course of individual life. Changes in bones also occur in evolution as functional adaptations to the environment. In this study, we report on the evolution of bone mass increase (BMI) that occurred in the postcranium and skull of extinct aquatic sloths. Although non-pathological BMI in postcranial skeleton has been known in aquatic mammals, we here document general BMI in the skull for the first time. We present evidence of thickening of the nasal turbinates, nasal septum and cribriform plate, further thickening of the frontals, and infilling of sinus spaces by compact bone in the late and more aquatic species of the extinct sloth *Thalassocnus*. Systemic bone mass increase occurred among the successively more aquatic species of *Thalassocnus*, as an evolutionary adaptation to the lineage's changing environment. The newly documented pachyostotic turbinates appear to have conferred little or no functional advantage and are here hypothesized as a correlation with or consequence of the systemic BMI among *Thalassocnus* species. This could, in turn, be consistent with a genetic accommodation of a physiological adjustment to a change of environment.

## 1. Background

It has been known since the nineteenth century that bone structure can change over the course of an individual's life in response to the pattern and magnitudes of mechanical loads and by other physiological processes [1,2]. While bone structure can adjust locally, for instance within an epiphysis (e.g. with exercise on the inclined surface [3]), within a bone (e.g. with tennis practice [4]) or in a region of the skeleton (e.g. with spaceflight under microgravity [5]), it has also been demonstrated that bone structure alteration can be systemic (relating to the entire organism). Indeed, Lieberman [6] showed experimentally that exercise can induce systemic cortical bone growth in pigs and armadillos, probably under the control of growth hormones.

Modifications of bone structure occur within evolutionary lineages of tetrapods (four-limbed vertebrates) as well (termed in this case evolutionary adaptations as opposed to those within the lifespan of an individual), probably as a result of selection for new lifestyles. For instance, the bones of flying species became lightweight [7], whereas those of shallow-diving aquatic species became heavier [8] (herein ‘aquatic’ will encompass both semi- and fully aquatic habits). The latter adaptation is associated with two forms of non-pathological bone mass increase (BMI), pachyostosis *sensu stricto* (increased morphological robustness) and osteosclerosis (increased bone density) [9]. BMI is the most commonly acquired bone structural adaptation to an aquatic environment, having evolved independently at least 20 times in tetrapods (amniotes reviewed by Houssaye [10]). The fact that BMI is such a common adaptation raises questions regarding which evolutionary mechanisms are involved in these acquisitions. BMI is viewed as a modification of bone structure in certain regions of the skeleton, probably as a functional adaptation for buoyancy and trim control [11]. The selection of a systemic bone structure alteration over the course of lineage diversification was hence never demonstrated. But it is noteworthy that a pachyosteosclerotic postcranium, along with a compact skull structure, including reduced but rather thick ethmoturbinates, are found in sirenians [11,12] (see further details in the Discussion).

Here, we use micro-computed tomography to investigate the endocranial structure of the extinct aquatic sloth *Thalassocnus*, known from the Neogene of western South America. The postcranial bone structure of this genus revealed the acquisition of an increasingly pronounced BMI from the earliest to the latest of its species, suggesting a progressive adoption of aquatic habits [13]. The skull, not routinely investigated in analyses of BMI, is here studied to (i) test if the BMI found in the postcranium of this genus also occurs in the cranium and (ii) explore other potential modifications of the endocranial anatomy that could be related to alterations of bone structure occurring during the evolution of this clade.

## 2. Material and methods

### (a) Specimens

A dataset was built to investigate the evolution of the endocranial structure of the aquatic sloth *Thalassocnus*. All sampled specimens of *Thalassocnus* are from the Pisco Formation, Peru. Three species of the genus were sampled: *T. natans* (7 Ma, *n* = 1), *T. littoralis* (6 Ma, *n* = 3) and *T. carolomartini* (5 Ma, *n* = 2) [14,15]. In *T. natans*, the postcranium exhibits incipient BMI, whereas in *T. littoralis* BMI is intermediate, and in *T. carolomartini* the BMI is most strongly expressed, suggesting a gradual adaptation to an aquatic lifestyle [13]. For comparison, several extinct terrestrial sloths were sampled (one specimen per taxon), including *Pelecyodon* (Santa Cruz Formation, Argentina, Early Miocene [16]), *Oreomylodon* (Punín, Ecuador, Late Pleistocene) and *Megatherium* (Río Salado, Argentina, Pleistocene). Both genera of extant sloths (*Bradypus* and *Choloepus*) and another extant pilosan (the clade that comprises sloths and anteaters), *Tamandua*, were also sampled (one specimen per taxon). See electronic supplementary material, additional file S1 for the catalogue numbers of all specimens. Imperfect preservation precluded the acquisition of measurements for some fossils (see electronic supplementary material, file S1), but qualitative observations were possible for all specimens. All sampled specimens are skeletally mature as indicated by suture closure (see also electronic supplementary material, file S2).

### (b) Data acquisition

The skulls of all specimens were scanned with micro-computed tomography at the AST-RX platform, Muséum national d'Histoire naturelle (MNHN), Paris, France (http://www.ums2700.mnhn.fr/ast-rx/ressources). Greyscale 16-bit stacks of .tif files were acquired with a resolution ranging from 0.05 to 0.14 mm (depending on the size of the specimens). All raw measurements are available in electronic supplementary material, file S1.

#### (i) Ethmoturbinate mean thickness and relative occupancy

We limited our quantitative analysis to the ethmoturbinates, because the more anteriorly positioned maxilloturbinates were not preserved in all specimens (nasoturbinates were not identified). But the maxilloturbinates, cribriform plate and nasal septum were also described qualitatively. The raw stacks were opened with the Fiji package [17] (combination of ImageJ, here ImageJ2 v. 1.51 g and plugins [18,19]) and reoriented (see electronic supplementary material, files S2, S3, figure S1D). A coronal section was selected just anterior to the level of the most posterior dorsal separation of the olfactory bulbs (electronic supplementary material, file S3, figure S1). A square (two-dimensional) region of interest (ROI), here referred to as the ‘ethmoid ROI’, was defined and thresholded (see electronic supplementary material, file S2). The ethmoturbinate mean thickness and its standard deviation (mm) were acquired with the ‘Thickness’ routine of the BoneJ plugin [20]. The ethmoturbinate relative occupancy of the nasal cavity at the level of the olfactory bulbs represents the proportion of surface of the ethmoid ROI occupied by bone (‘Measure’ routine of the base of Fiji).

#### (ii) Skull roof compactness

A ‘skull roof’ ROI was defined to represent the overall compactness of the skull roof (see electronic supplementary material, files S2, S3 and figure S1D). The skull roof compactness was then measured within the skull roof ROI as for the ethmoturbinate relative occupancy of the ethmoid ROI (after thresholding; see above).

#### (iii) Cranial vault thickness

Using the oriented CT-scan stack (see above and electronic supplementary material, files S2, S3, figure S1A–C), we measured with Fiji the thickness of the cranial vault (in mm) at five standard locations, adapting the definitions of Lieberman [6]: supraoccipital anteromedial corner (for further precision, we selected the point of least thickness); maximum thickness of the nuchal region in the midsagittal plane; middle of the parietal (Lieberman [6] used the parietal eminence, but this structure is absent in pilosans [21]); bregma (average of parietal and frontal) and centre of the frontal along the midsagittal axis (the measure was taken internally up to the dorsalmost level of the frontal sinus on the corresponding coronal section).

### (c) Statistical analysis

Data analysis was performed with RStudio v. 1.0.153 (using R v. 3.4.1 [22]). For each parameter, a correlation with body size was assessed using phylogenetic generalized least-squares linear regressions (gls function, nlme package [23]) with a within-group correlation structure based on the optimized phylogenetic signal index lambda (with the corPagel function, ape package [24]; see [25]). As a size proxy, we used tooth row length (from the mesial side of M1 to the distal side of M4), because it could be measured even on fragmentary specimens. For species represented by several specimens, the mean of each measurement was used.

Two timetrees were used for the phylogenetically informed analysis, representing the current main hypotheses regarding sloth phylogeny (see electronic supplementary material, file S2). The function contMap (phytools package REF) was used to map values of the skull roof compactness (using maximum likelihood for reconstruction of the ancestral states) onto the timetree. Graphics were produced with strap [26], ggplot2 [27], reshape [28] and cowplot [29] packages.

## 3. Results

### (a) Ethmoturbinate thickness and relative occupancy

**Figure 1.**
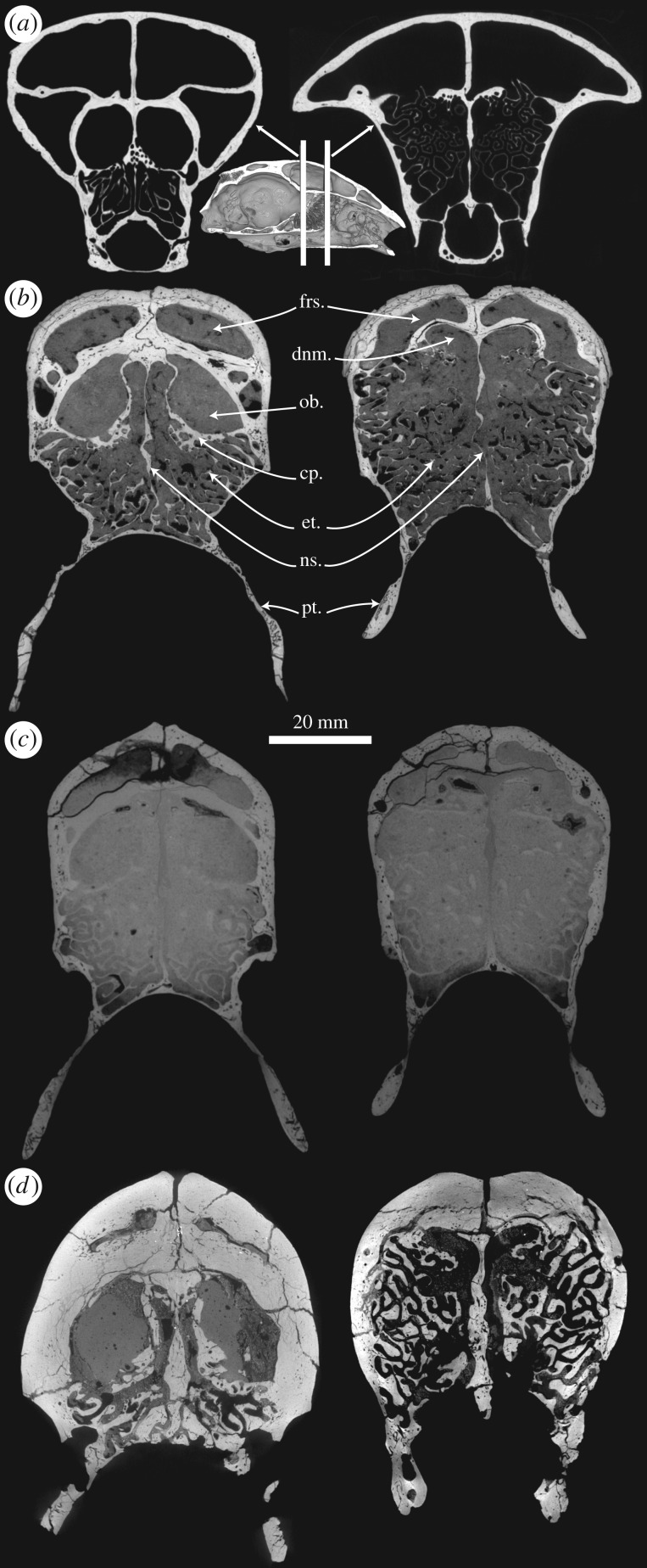
Evolution of cranial pachyosteosclerosis in the aquatic sloth *Thalassocnus*. Coronal sections (virtual) of the skull at the level of the posterior region of the olfactory bulbs (left) and just anterior to them (right). (*a*) Extant sloth *Choloepus didactylus* (MNHN.ZM-MO1996-594). A three-dimensional rendering of a parasagittally (virtually) sliced skull in medial view is displayed to locate the selected coronal sections. (*b*) *Thalassocnus natans* (MNHN.F.SAS734; postcranially non-pachyostotic and incipiently osteosclerotic). (*c*) *Thalassocnus littoralis* (MNHN.F.SAS1615; postcranially of intermediate pachyosteosclerosis). (*d*) *Thalassocnus carolomartini* (SMNK-3814; postcranially strongly pachyosteosclerotic). Abbreviations: cp., cribriform plate; dnm., dorsal nasal meatus; et., ethmoturbinates; frs., frontal sinus; ns., nasal septum; ob., olfactory bulb endocast; pt., pterygoid. The scale does not apply to the smaller three-dimensional rendering of the skull in (*a*). See also electronic supplementary material, files S4–S6.

**Figure 2.**
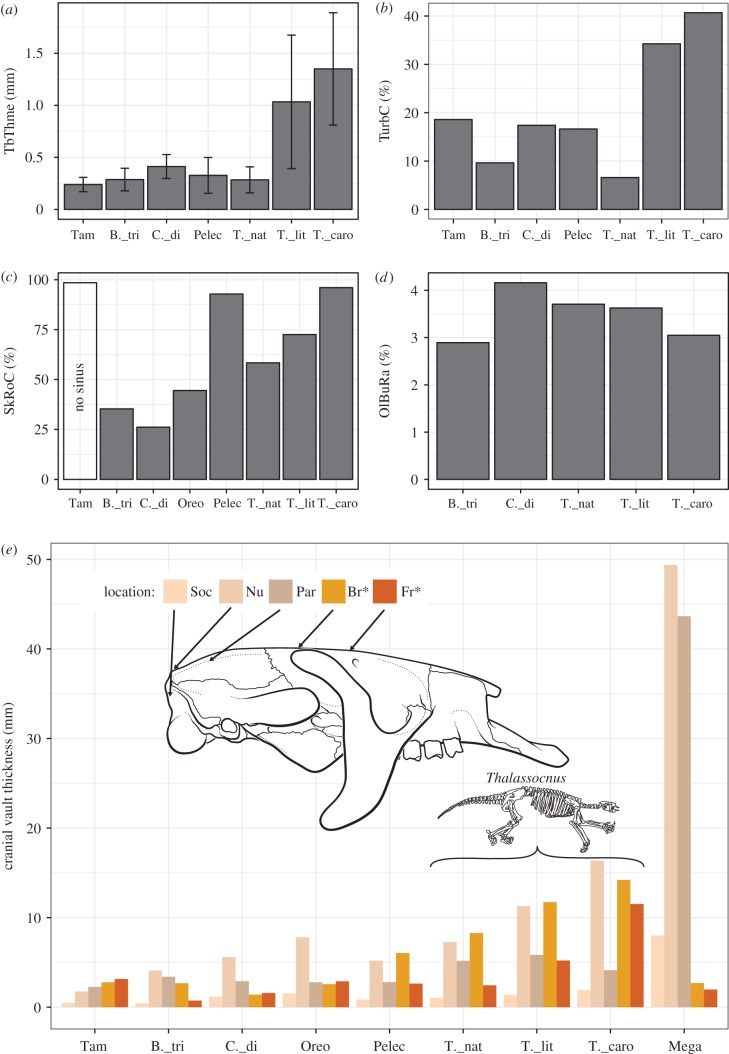
Quantification of the cranial pachyosteosclerosis and olfactory bulb volume ratio in pilosans (anteater and sloths). (*a*) Ethmoturbinate mean thickness (TbThme), as measured in the ethmoid region of interest (ethmoid ROI; see electronic supplementary material, file S3, figure S1D). Error bars indicate standard deviation. See also figure 3. (*b*) Ethmoturbinate relative occupancy (TurbC). Proportion of the ethmoid ROI surface occupied by bone. (*c*) Skull roof compactness (SkRoC) as measured in the skull roof ROI (see electronic supplementary material, file S3, figure S1D). The anteater *Tamandua* differs from sloths in the absence of frontal sinuses, biasing its value. (*d*) Ratio of olfactory bulbs endocast to total brain endocast volume (OlBuRa). (*e*) Skull vault thickness at standard locations, as represented in a drawing in the right lateral view of a skull of *Thalassocnus*. Locations with asterisk indicate no significant correlation with body size (as shown by linear regression *p* > 0.05). Location abbreviations: Soc, supraoccipital anteromedial corner; Nu, maximum thickness of the nuchal region; Par, parietal; Br, bregma; Fr, centre of the frontal. Species abbreviations: B._tri, *Bradypus tridactylus*; C._di, *Choloepus didactylus*; Mega, *Megatherium*; Oreo, *Oreomylodon*; Pelec, *Pelecyodon*; Tam, *Tamandua*; T._caro, *Thalassocnus carolomartini*; T._lit, *T. littoralis*; T._nat, *T. natans*. See also electronic supplementary material, files S1, S3.

**Figure 3.**
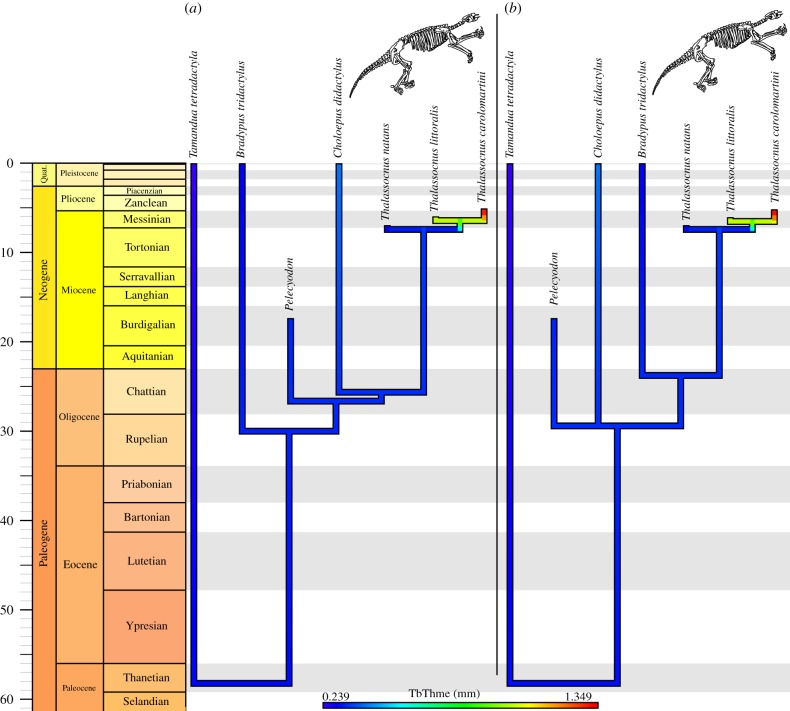
Timetrees with mapped ethmoturbinate mean thickness. (*a*) Morphological time-calibrated phylogeny, based on [14,15,30,31]. (*b*) Mitochondrial time-calibrated phylogeny, primarily based on [32]. See also electronic supplementary material, files S1, S3, figure S1. (Online version in colour.)

In all studied taxa, including the latest species of *Thalassocnus*, we observed well-developed and complex maxillo- and ethmoturbinates, the latter filling the whole ethmoid region of the nasal cavity. In non-aquatic pilosans (extant anteaters and sloths; figure 1*a*; electronic supplementary material, file S4; and extinct sloths) and the early species of *Thalassocnus* (*T. natans*, figure 1*b*; electronic supplementary material, file S5), the ethmoturbinates are thin, scrolled plates of bone (approx. 0.3 ± 0.1 mm thick; figure 2*a*; electronic supplementary material, file S1; see the Material and methods section for details on the measurements), as is generally the case in terrestrial and semiaquatic mammals. Accordingly, the ethmoturbinate relative occupancy (proportion of the area of the ethmoid ROI occupied by bone) is roughly 10–19% (figure 2*b*). By contrast, in the later species of *Thalassocnus* (figure 1*c*,*d*; electronic supplementary material, file S6), the ethmoturbinates are thickened (exceeding 1 mm of mean thickness, up to 1.5 ± 0.7 mm in *T. carolomartini*; figure 2*a*), forming, especially in the latest species, heavy scrolls that occupy roughly 40% of the area (figure 2*b*). A clear increase in ethmoturbinate mean thickness is apparent between the ancestral condition reconstructed for *Thalassocnus* and the most recent species of the genus (figure 3). Although not quantified, it can be stated that the maxilloturbinates (nasoturbinates not identified), cribriform plate and nasal septum are also conspicuously thickened in the latter species (figure 1*d*; electronic supplementary material, file S6).

### (b) Skull roof compactness and cranial vault thickness

In the early species of *Thalassocnus*, *T. natans* (figure 1*b*), as in all arboreal (figure 1*a*; electronic supplementary material, files S4, S5) and terrestrial sloths (and many other mammals [33]), the skull roof is pneumatized due to the presence of one or several well-developed frontal sinuses. The walls of the skull vault show a slight porosity, with regions of spongy bone present. Measured on a coronal section at the level of the posterior end of the olfactory bulbs, the skull roof compactness in these taxa is approximately 41% (except for the terrestrial sloth *Pelecyodon* that features an exceptionally small sinus; figure 2*c*; electronic supplementary material, file S1). In both later species of *Thalassocnus* (*T*. *littoralis* and *T. carolomartini*), the frontal sinus is partly filled with compact bone (figures 1*d*; electronic supplementary material, file S3, figure S2, file S6). Consequently, skull roof compactness of these species is increased, reaching an average of 96% for *T. carolomartini* (figures 2*c*; electronic supplementary material, file S3, figure S2). The skull vault walls are also almost entirely composed of compact bone (i.e. with virtually no spongy bone) and generally thicker than in the early species. Cranial vault thickness (CVT) is also increased at all locations (except for the parietal eminence) in the two later species of *Thalassocnus* (figure 2*e*). At the regions of the bregma and at the middle of the frontal, skull thickness approaches 15 mm in the latest species of *Thalassocnus*, which is over five times thicker than that of the elephant-sized terrestrial sloth *Megatherium* at the same locations. The walls of the posterior region of the skull are rather thick in all sloths, probably due to mechanical demands (i.e. cervical muscles attachment sites), and are influenced by size (probably tying to weight of the skull). Indeed, skull vault thickness is significantly correlated with size only at the supraoccipital, nuchal region and parietal eminence (linear regressions *p* < 0.05). Nevertheless, in non-aquatic pilosans (anteaters and arboreal and terrestrial sloths; electronic supplementary material, file S4) and the early species of *Thalassocnus* (*T. natans*; electronic supplementary material, file S5), the walls of the posterior region (e.g. at the external occipital protuberance) have a diploë structure (thin inner and outer layers of compact bone sandwiching a spongy core [34]). This differs from the later species of *Thalassocnus* (*T. carolomartini*, electronic supplementary material, file S6), in which there is minimal or no spongy bone present, confirming that the structure of the whole skull is more compact.

## 4. Discussion

As has been documented for the postcranial skeleton [13,35], the skull of the aquatic sloth *Thalassocnus* exhibits gradual BMI from the earliest to the latest of its species. The earliest-sampled species of *Thalassocnus*, *T. natans*—without postcranial osteosclerosis and pachyostosis and supposedly less aquatic—shows an endocranial structure similar to that of terrestrial pilosans, and mammals in general (i.e. paper-thin turbinates and a highly pneumatized skull roof, with the presence of large frontal sinuses). The two later species, *T. littoralis* and especially *T. carolomartini*, display postcranial osteosclerosis (and pachyostosis in the latter) and are assumed to have been more aquatic than *T. natans*. Both also have strongly thickened (pachyostotic) turbinates and osteosclerotic skulls characterized by a thick skull roof with a high proportion of compact to spongy bone and infilling of the frontal sinuses with compact bone. BMI hence affected both dermal (e.g. frontal) and endochondral bones (e.g. supraoccipital). The measured parameters were not significantly correlated with body size (except for skull vault thickness in the posterior region), indicating that the modifications of cranial structure among the species of *Thalassocnus* are not driven by changes in body size.

Endocranial anatomy is known for only two other groups that acquired conspicuous osteosclerosis and/or pachyostosis: the sirenians and early cetaceans. The skulls of modern adult sirenians are composed largely of compact bone, but trabecular bone persists in some places (particularly in the rostrum [11]). Extant sirenians have a rudimentary olfactory system, with greatly reduced olfactory bulbs and reduced turbinates [12,36]. The latter are quite robust and thick (no quantitative assessment was made). In the earliest-diverging sirenian *Prorastomus* (Early Eocene [37]), the skull walls are thick and compact, and the ethmoturbinates are described as more extensive and thinner than in the later members of the clade [38]. In *Protosiren* (Middle Eocene; see [39], fig. 4.083), the skull walls are similar, and the ethmoturbinates appear to be rather thick but not as reduced as in extant sirenians. However, no turbinates (including maxillo- and nasoturbinates) seem to have extended anterior to the ethmoid region (see [39], fig. 4.080–86). It is noteworthy that the ethmoturbinates of *Protosiren* seem thinner and less well developed than in *T. carolomartini* (in which maxilloturbinates are well developed). Other early sirenians, such as *Libysiren* [40] and *Eotheroides* [41], also show less of a reduction of their ethmoturbinates than extant sirenians. Reduction of the olfactory bulbs occurred early in the evolutionary history of sirenians (e.g. *Protosiren* [39] and *Eotheroides* [41]). The earliest sirenian for which cranial and postcranial material is associated is *Pezosiren* (lower Middle Eocene). This taxon, one of the oldest known sirenians, already had strong BMI, as in most of the later members of the clade [9]. It therefore appears likely that, in contrast to *Thalassocnus*, sirenians acquired strong BMI early in their history, along with a reduction of the olfactory bulbs and a simplification of the turbinates. However, the rather thick turbinates of the later diverging *Protosiren* [39] (and reduced but quite robust turbinates of extant sirenians) suggest that a thickening analogous to that of *Thalassocnus* might have occurred in the very early history of sirenians. Although the successively more aquatic sloths and sirenians show broadly similar evolutionary modification of bones, the two lineages also show adaptive differences and their convergence is incomplete. It appears that the endocranial characters of *Thalassocnus* during the initial stage of the evolutionary adaptation of the *Thalassocnus* lineage are more precociously developed and not present to the same degree in any known sirenian.

In extant cetaceans (which notably differ from *Thalassocnus* and sirenians in being active swimming predators), the olfactory system is greatly reduced, with a nearly complete or complete loss of the turbinates, cribriform plate and olfactory bulbs [42]. In the earliest members of the clade for which data are available, such as the ‘archaeocete’ *Remingtonocetus* (Middle Eocene), the skull is highly transformed, with an elongate nasal cavity apparently mostly devoid of turbinates (at least anterior to the level of P4). On the whole, the earliest cetaceans for which we have data already show a strong reduction of the turbinates when compared with the general condition of terrestrial mammals, and show osteosclerotic postcrania (BMI is even found in the raoellid *Indohyus* [43]), in which the ribs may also be pachyostotic [44–46] (see a more detailed discussion on cetaceans in electronic supplementary material, file S7).

To our knowledge, the endocranial structure of other aquatic mammal lineages with conspicuous postcranial BMI, such as desmostylians, has never been documented. In other aquatic mammals that did not acquire conspicuous osteosclerosis or pachyostosis (either postcranial or cranial), the olfactory system is often reduced to some extent (i.e. the platypus, carnivorans, tenrecids, soricids and talpids [47]), and, to our knowledge, no thickening of the turbinates has been documented. In the sea otter (*Enhydra lutris*), the ribs are viewed as incipiently osteosclerotic [9] and the long bones exhibit BMI [48]. This taxon's ‘olfactory turbinates' (ethmoturbinates and nasoturbinates) are reduced [49], but the turbinates do not appear to be thickened when compared to those of other carnivorans. BMI is also documented in the ribs and long bones of the platypus (*Ornithorhynchus* [50,51]), which also displays a reduction of the olfactory system [47] without any conspicuous thickening of the turbinates (E.A. 2017, personal observation; AMNH 200255 on digimorph.org).

The endocranial structure in the late species of the aquatic sloth *Thalassocnus* is hence unique in that they retain well-developed maxillo- and ethmoturbinates that are dramatically inflated (pachyostotic). The ethmoturbinates were most likely functional, probably bearing olfactory epithelium, because (i) the cribriform plate has essentially conserved its configuration and (ii) the olfactory bulbs are well developed (figure 2*d*; see also electronic supplementary material, files S3, S7, figure S3; [52]). Thus, their sense of smell was possibly comparable to that of extant sloths (which are macrosmatic [53]).

The reduction of frontal sinus size in aquatic sloths could have had functional implications. However, no clear functional understanding of the loss of the frontal sinus has been reached in other mammals, as it is well documented in arctoid carnivorans [54] (more generally, the function of the frontal sinus is poorly understood [33]). The lack of paranasal sinuses was suggested to be an adaptation to diving in extant cetaceans (to avoid fracture of the sinus walls caused by pressure variations [55]), but this can hardly be argued for sirenians or aquatic sloths, which are/were likely shallow divers, as shown for the latter by their strong osteosclerosis and pachyostosis [9,13]. It is not evident, either, how cranial BMI would function as buoyancy and trim control [56,57], as assumed for postcranial BMI [11]. Pachyostotic turbinates seem to be even more challenging to interpret functionally, as the key function of the turbinates is to provide an expanded surface area for the olfactory and respiratory mucosa and in some cases to guide airflow [58]. One can hardly argue that pachyostotic turbinates provide a significant gain of mass, so a role in the buoyancy and trim control would be at best minimal. Furthermore, hypertrophic turbinates are sometimes associated in humans with pathological conditions [59]. It is difficult to categorically rule out that pachyostotic turbinates were beneficial in some way, but if they did not confer greater fitness, it appears more parsimonious to interpret pachyostotic turbinates as the result of a systemic process of BMI affecting the skeleton of the late species of *Thalassocnus*. As a corollary, the aquatic sloth's pachyostotic turbinates can be viewed as an evolutionary by-product (or ‘evolutionary spandrel’ [60]) of its adaptation to shallow diving.

It is hence shown here for the first time that systemic bone mass alteration, formerly known only as a physiological adjustment [6], was probably selected as an evolutionary adaptation. Other lineages that have acquired similar adaptations to the aquatic environment seem to show a lesser integration of their bone structure. However, the rather thick turbinates of the early sirenian *Protosiren* (and possibly the reduced but quite robust turbinates of extant sirenians) might suggest that systemic BMI acquisition has potentially occurred in other clades. The skeleton of one of the earliest sirenians, *Pezosiren*, is already affected by conspicuous BMI, which further shows that the earliest stage of their adaptation to the aquatic environment is probably undocumented, and might have also been marked by the acquisition of a systemic BMI. Unlike the case of sirenians, the extremely detailed fossil record of *Thalassocnus* appears to document a very early stage in the process of adaptation to an aquatic environment, allowing us to grasp the relationship between what could have initially been a physiological adjustment and the evolutionary adaptation of bone structure.

In this new light, previous incongruences can be better understood. For the extinct sirenian *Corystosiren*, it was stated that ‘the striking thickness of its skull roof […] has no obvious functional explanation’ ([57], p. 369). As stated above, sirenians acquired pachyosteosclerosis to some extent, and the endocranial structure of this taxon might also be the result of a systemic process; however, in their case a functional explanation could also be considered, as loading by compressive forces may be transmitted through the dorsum of the skull from the premaxilla-frontal joints during tusk use [61]. In early humans, Pleistocene species of *Homo* in particular, the cause of high CVT is still highly debated with possible causes being protection from injury, response to local loading, diet, locomotion, variation in geomagnetism or part of a systemic process [6,62]. A recent analysis showed that it is a combination of high CVT and a relatively thick inner spongy region (therein called diploë) that characterizes some early hominins [63]. Similarly, according to the latter analysis, the cause of such a cranial vault structure in early humans is not yet understood. Our results emphasize the importance of taking into account postcranial bone structural adaptations when interpreting cranial data. More generally, an understanding of the overall structural adaptations of a taxon's skeleton should preferably be gained before drawing functional interpretations.

## 5. Conclusion

Systemic bone structure alteration, formerly known exclusively as a physiological adjustment, was here evidenced to have been retained as an evolutionary adaptation thanks to the outstandingly detailed (both in terms of geological age and anatomy) and early-stage record of a land-to-sea transition in the extinct sloth *Thalassocnus*. This new result is consistent with a macroevolutionary process of selection on environmentally induced variation of phenotypic plasticity [64–69]. In other words, the systemic alteration of the highly plastic bone structure that gradually evolved among the species of *Thalassocnus* may represent an example of a macroevolutionary transition from a phenotypic accommodation (to an environmental change) to a genetic accommodation. In the context of the so-called extended (evolutionary) synthesis, Pigliucci [70] points out the difficulty of uncovering such examples, which are required to corroborate the hypothesis that phenotypic plasticity has an important macroevolutionary role.

The precise mechanism causing an adjustment (over the course of an individual's life) of bone structure in response to life in water is not understood. However, one can speculate that the shift of a terrestrial animal to an aquatic environment involves an increase in exercise intensity, which was shown to induce BMI [6,71]. There does not seem to be a clear influence of swimming on the bone mass of athletes [72] (but differences in other physical activities probably prevent a direct correlation assessment in humans [73]). However, rats that were swim-trained during growth have a greater overall bone mineral content and bone surface than the sedentary controls [74] (but bone mineral density did not differ; and see [75] for the opposite effect of endurance swim training on trabecular bone). It is noteworthy, however, that swim-trained animals do not necessarily experience a greater overall exercise intensity, as they were found to voluntarily run less outside the experimental exercise than control and running groups [74]. Furthermore, bone structure at locations not directly influenced by locomotion, such as the cranial vault, does not seem to have been investigated in swim-trained animals.

The lack of a similarly detailed fossil record in other ancestrally terrestrial tetrapods adapted to an aquatic lifestyle prevented drawing such a conclusion in their respective cases, but a similar process might have occurred during the evolutionary history of at least some of them. BMI is probably the most widespread lifestyle adaptation among aquatic tetrapods. This suggests that genetic accommodation of a trait subject to physiological adjustment such as bone structure alteration might have played an important role in great evolutionary transitions, of which the secondary adaptations of tetrapods to an aquatic lifestyle is an iconic example.

## Supplementary Material

Raw data table

## Supplementary Material

Additional Material and Methods

## Supplementary Material

Supplementary Figures

## Supplementary Material

Additional Results and Discussion

## Data accessibility

All data generated and directly analysed during this study are included in this published article and are deposited in the Dryad Digital Repository (https://doi.org/10.5061/dryad.7gq2tb0) [76]. Further data that relate to the original CT-scan images, as well as the three-dimensional models and their reconstruction, are also available: (i) the CT-scan images of the anatomical regions analysed in this study are available upon request to the curators of the MNHN fossil mammal collections; (ii) three-dimensional virtual reconstruction data generated and analysed during the current study (brain endocasts) are available in MorphoMuseuM: http://dx.doi.org/10.18563/journal.m3.64 [52].
