# Supplementary material for: Evolutionary adaptation to aquatic lifestyle in extinct sloths can lead to systemic alteration of bone structure
Source: Proc Biol Sci. 2018 May 9;285(1878):20180270. doi: 10.1098/rspb.2018.0270 (PMC5966604; doi:10.1098/rspb.2018.0270)
Supplement: Additional Material and Methods [file rspb20180270supp2.pdf]

Evolutionary Adaptation to Aquatic Lifestyle in Extinct Sloths Can Lead to Systemic Alteration of Bone Structure

Eli Amson[1,2,3,*], Guillaume Billet[4], and Christian de Muizon[4].

[1]*Museum für Naturkunde, Leibniz-Institut für Evolutions- und Biodiversitätsforschung, Invalidenstraße 43; Berlin; 10115; Germany*
[2]*AG Morphologie und Formengeschichte, Institut für Biologie; Humboldt Universität zu Berlin, Philippstraße 13; Berlin; 10115; Germany*
[3]*Bild Wissen Gestaltung. Ein Interdisziplinäres Labor; Humboldt Universität zu Berlin, Sophienstraße 22a; Berlin; 10178; Germany*
[4]Centre de Recherche sur la Paléobiodiversité et les Paléoenvironnements-CR2P (CNRS, MNHN, UPMC, Sorbonne Universités), Département Origines et Évolution; Muséum national d'Histoire naturelle, 8 rue Buffon; Paris; 75005; France
[*]Correspondence to be sent to: *Humboldt Universität zu Berlin; Unter den Linden 6; 10099; Berlin; Germany. eli.amson@mfn.berlin*

Additional file 2: Supplementary Material and Methods

Specimens

See main text and the following:

Specimens ontogenetic stage

All sampled specimens are skeletally mature, as indicated by cranial sutures closure. One should not interpret the opened basicranial and sagittal sutures of the specimen *T. carolomartini* SMNK-3814 (Figure 1a, electronic supplementary material, additional file 6) as indicative of an early ontogenetic stage, as this is due to taphonomy and preservation. Other cranial sutures of this specimen are closed (see additional file 6; the frontal-frontal suture for instance is one of the first to close in extant sloths [1]). Furthermore, fully articulated hands were associated with the skull, and show complete fusion of the epiphyses [1: Fig. 17]. Similarly, the specimen of the same species MNHN.F.SAO203 is associated with a femur with thorough epiphyseal fusion (data from [3]).

Data acquisition

For general information about the scans, see main text.

Ethmoturbinate mean thickness and relative occupancy

Orientation—The skulls were orientated with successive 'Re-slicing' routines: the sagittal plane was set along the Z-axis, the mediolateral plane along the X-axis, and the dorsoventral plane (as given by a plane normal to the anteroposterior lengthening of the palate) along the Y-axis. A coronal section was selected just anterior to the level of the most posterior dorsal separation of the olfactory bulbs (see electronic supplementary material, additional file 3, Figure S2).

Ethmoid ROI— A square (two-dimensional) region of interest (referred to as 'ethmoid ROI' herein; electronic supplementary material, additional file **3**, Figure S**2**D) was defined to be centred mediolaterally relative to centre of the olfactory bulb, centred dorsoventrally relative to the nasal cavity, and as large as possible but fitting within the nasal cavity (to comprise proximal turbinates that are mostly covered by olfactory epithelium, see [4,5] and references therein). A 3D approach was precluded by the preservation of the fossils, including in some places sediment as dense as the bones. If present, parts of cribriform plate were removed (for the fossil of *Pelecyodon*, the ethmoid ROI placement was approximate, due to the preservation of the specimen). The obtained slice was thresholded (which for some fossils required prior deletion of sedimentary matrix) with the 'Optimise Threshold> Threshold Only' routine of the BoneJ plugin [6]. For one specimen with particularly thin ethmoturbinates (*Tamandua tetradactyla* 2_3.7.7.135), the image was sharpened ('Sharpen' routine of base of Fiji) prior to thresholding.

Thickness measurement— The ethmoturbinate mean thickness and its standard deviation (mm) were acquired with the 'Thickness' routine of BoneJ [6]. Because it is measured on a 2D coronal section, this measurement represents approximate local turbinal thickness. The lengthening of the ethmoturbinates being arranged similarly across the sampled taxa (proximoldistally, along the Z-axis), it should not overly bias our observations (but greater accuracy would be obtained with a 3D ROI). The ethmoturbinate relative occupancy of the nasal cavity at the level of the olfactory bulbs represents the proportion of surface of the ethmoid ROI occupied by bone ('Measure' routine of the base of Fiji).

Skull roof compactness

Skull roof ROI—A region of interest (referred to as 'skull roof ROI' herein) on the coronal section previously defined (see above and electronic supplementary material, additional file 3, Figure S2) was defined to represent the overall compactness of the skull roof. The skull roof ROI comprises the frontal bone, limited ventrally at the mid-depth (dorsoventral) of the olfactory bulb's cavity, and excluding the ethmoid bone located ventral to the dorsal edge of the olfactory bulb's cavity (see above and electronic supplementary material, additional file 3, Figure S2D). For some fossil specimens, cracks had to be deselected to avoid overestimating the porosity.

Cranial vault thickness (CVT)

The same skull orientation was used as for the other measurements (see above). See main text for further details on the acquisition of the CVT measurements.

Brain endocast

Using the raw CT-scan stacks (see above), the brain endocasts were segmented using Mimics (Materialize) or Avizo (FEI Visualization science group) softwares. Surface models were exported in STL format, and then converted to OBJ format using the software Meshlab [7]. Geomagic Studio (3D Systems) was then used to clean the models, i.e, trimming of the spinal cord endocast and vessels and repairing of holes, in order to obtain a watertight model. Small portions of a fragmentary specimen (*T. carolomartini*, SMNK-3814) had to be completed as well (see electronic supplementary material, additional file 3, Figure S333C). Total brain endocast volume and that of each olfactory bulb (see selection in electronic supplementary material, additional file 3, Figure S33) was then measured (both in mm^3). The ratio of both olfactory bulbs endocast volume to that of the whole brain was finally computed. For one fossil specimen (*Thalassocnus carolomartini* SMNK3814), only one bulb could have been segmented, so its volume was doubled.

STATISTICAL ANALYSIS

See main text and the following:

Timetrees

Two timetrees were used for the phylogenetically informed analysis, representing the current main hypotheses regarding sloths' phylogeny. For the 'morphological time-calibrated phylogeny', we used as a base the results of Gibb et al. [8]. The tree was modified with the phytools package (bind.tip function; [9]) to include extinct sloths according to previous phylogenetic hypotheses and dating ([10–12]; length of the branches leading to nodes of unknown ages, which are in direct relation to extinct taxa, and from these to terminal extinct taxa, were arbitrarily set at 1 and 0.1 Myr, respectively). Furthermore, the split between Mylodontidae (represented by *Oreomylodon*) and the other non-*Bradypus* sloths was set according to the age of the oldest fossil pertaining to the clade (Deseadan) [13]. The same was applied for the *Megatherium*-*Thalassocnus* divergence (Santacrucian) [10]. For the 'mitochondrial time-calibrated phylogeny', the timetree of Slater et al. [14] was used as a base, and the extinct taxa were added as described above (*Thalassocnus* and *Nothrotheriops* both

being included in Megatheria [10], the latter was replaced by the former; both *Oreomylodon* and *Mylodon* being mylodontids, the latter was replaced by the former; the position of *Pelecyodon* not being deducible from Slater et al.'s topology, it was placed in a basal polytomy). Because *Tamandua* was not included in Slater et al. [14], its age of divergence with sloths was set according to Gibb et al. [8].

References

1. Rager L, Hautier L, Forasiepi AM, Goswami A, Sánchez-Villagra MR. 2014 Timing of cranial suture closure in placental mammals: Phylogenetic patterns, intraspecific variation, and comparison with marsupials. *J. Morphol.* **275**, 125–140. (doi:10.1002/jmor.20203)
2. Amson E, Argot C, McDonald HG, Muizon C de. 2015 Osteology and functional morphology of the forelimb of the marine sloth *Thalassocnus* (Mammalia, Tardigrada). *J. Mamm. Evol.* **22**, 169–242. (doi:10.1007/s10914-014-9268-3)
3. Amson E, Argot C, McDonald HG, de Muizon C. 2015 Osteology and Functional Morphology of the Hind Limb of the Marine Sloth Thalassocnus (Mammalia, Tardigrada). *J. Mamm. Evol.* **22**. (doi:10.1007/s10914-014-9274-5)
4. Pihlström H. 2008 Comparative Anatomy and Physiology of Chemical Senses in Aquatic Mammals. In *Sensory Evolution on the Threshold Adaptations in Secondarily Aquatic Vertebrates* (eds JGM Thewissen, S Nummela), pp. 95–109. Berkeley: University of California Press. (doi:10.1525/california/9780520252783.003.0007)
5. Van Valkenburgh B, Curtis A, Samuels JX, Bird D, Fulkerson B, Meachen-Samuels J, Slater GJ. 2011 Aquatic adaptations in the nose of carnivorans: evidence from the turbinates. *J. Anat.* **218**, 298–310. (doi:10.1111/j.1469-7580.2010.01329.x)
6. Doube M, Kłosowski MM, Arganda-Carreras I, Cordelières FP, Dougherty RP, Jackson JS, Schmid B, Hutchinson JR, Shefelbine SJ. 2010 BoneJ: Free and extensible bone image analysis in ImageJ. *Bone* **47**, 1076–1079. (doi:10.1016/j.bone.2010.08.023)
7. Cignoni P, Callieri M, Corsini M, Dellepiane M, Ganovelli F, Ranzuglia G. 2008 MeshLab: an Open-Source Mesh Processing Tool. In *Eurographics Italian Chapter Conference* (eds V Scarano, R De Chiara, U Erra), The Eurographics Association. (doi:10.2312/LocalChapterEvents/ItalChap/ItalianChapConf2008/129-136)
8. Gibb GC, Condamine FL, Kuch M, Enk J, Moraes-Barros N, Superina M, Poinar HN, Delsuc F. 2016 Shotgun mitogenomics provides a reference phylogenetic framework and timescale for living xenarthrans. *Mol. Biol. Evol.* **33**, 621–642. (doi:10.1093/molbev/msv250)
9. Revell LJ. 2012 phytools: An R package for phylogenetic comparative biology (and other things). *Methods Ecol. Evol.* **3**, 217–223. (doi:10.1111/j.2041-210X.2011.00169.x)
10. Amson E, De Muizon C, Gaudin TJ. 2016 A reappraisal of the phylogeny of the megatheria (Mammalia: Tardigrada), with an emphasis on the relationships of the Thalassocninae, the marine sloths. *Zool. J. Linn. Soc.* **179**. (doi:10.1111/zoj.l2450)
11. Muizon C de, McDonald HG, Salas R, Urbina M. 2004 The youngest species of the aquatic sloth *Thalassocnus* and a reassessment of the relationships of the nothrothere sloths (Mammalia: Xenarthra). *J. Vertebr. Paleontol.* **24**, 387–397. (doi:10.1671/2429a)
12. Ehret DJ, Macfadden BJ, Jones DS, DeVries TJ, Foster DA, Salas-Gismondi R. 2012 Origin of the white shark *Carcharodon* (Lamniformes: Lamnidae) based on recalibration of the Upper Neogene Pisco Formation of Peru. *Palaeontology* **55**, 1139–1153. (doi:10.1111/j.1475-4983.2012.01201.x)

13. Pujos F, De Iuliis G. 2007 Late Oligocene Megatherioidea fauna (Mammalia: Xenarthra) from Salla-Luribay (Bolivia): new data on basal sloth radiation and Cingulata-Tardigrada split. *J. Vertebr. Paleontol.* **27**, 132–144.
14. Slater GJ, Cui P, Forasiepi AM, Lenz D, Tsangaras K, Voirin B, de Moraes-Barros N, MacPhee RDE, Greenwood AD. 2016 Evolutionary relationships among extinct and extant sloths: The evidence of mitogenomes and retroviruses. *Genome Biol. Evol.* **8**, 607–621. (doi:10.1093/gbe/evw023)
