# Supplementary material for: Evolutionary adaptation to aquatic lifestyle in extinct sloths can lead to systemic alteration of bone structure
Source: Proc Biol Sci. 2018 May 9;285(1878):20180270. doi: 10.1098/rspb.2018.0270 (PMC5966604; doi:10.1098/rspb.2018.0270)
Supplement: Supplementary Figures [file rspb20180270supp3.pdf]

# Evolutionary Adaptation to Aquatic Lifestyle in Extinct Sloths Can Lead to Systemic Alteration of Bone Structure

Eli Amson[1,2,3,*], Guillaume Billet[4], and Christian de Muizon[4].

[1]*Museum für Naturkunde, Leibniz-Institut für Evolutions- und Biodiversitätsforschung, Invalidenstraße 43; Berlin; 10115; Germany*
[2]*AG Morphologie und Formengeschichte, Institut für Biologie; Humboldt Universität zu Berlin, Philippstraße 13; Berlin; 10115; Germany*
[3]*Bild Wissen Gestaltung. Ein Interdisziplinäres Labor; Humboldt Universität zu Berlin, Sophienstraße 22a; Berlin; 10178; Germany*
[4]Centre de Recherche sur la Paléobiodiversité et les Paléoenvironnements-CR2P (CNRS, MNHN, UPMC, Sorbonne Universités), Département Origines et Évolution; Muséum national d'Histoire naturelle, 8 rue Buffon; Paris; 75005; France
[*]Correspondence to be sent to: *Humboldt Universität zu Berlin; Unter den Linden 6; 10099; Berlin; Germany. eli.amson@mfn.berlin*

# Additional file 2: Supplementary Figures

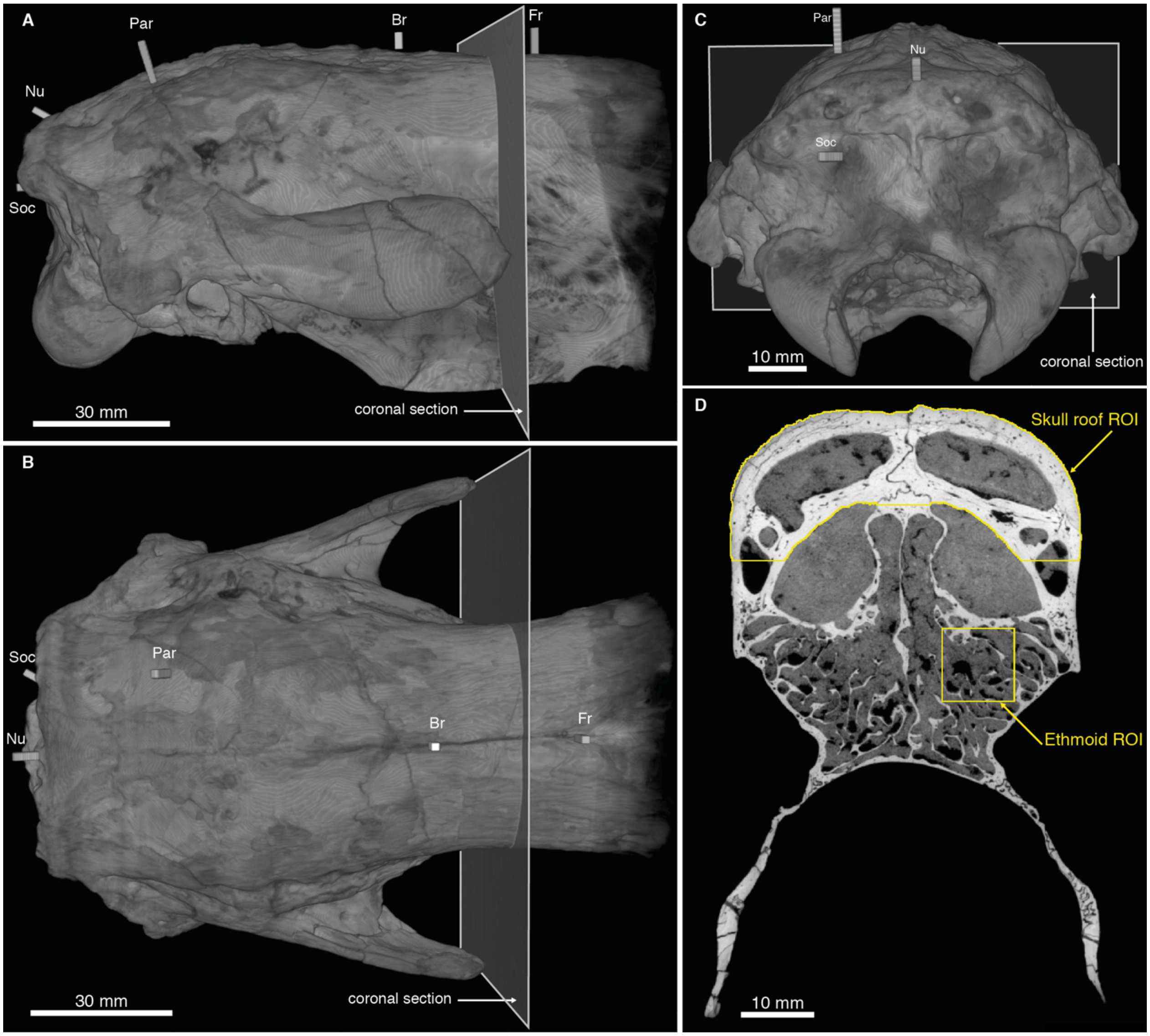


**Figure S1. Standard locations of measurement of cranial vault thickness and definition of the regions of the regions of interest (ROIs). The skull (without the anterior part) of *Thalassocnus natans* (MNHN.F.SAS734) is shown as an example. Related to Figures 2-3.**
(A) Right lateral view of the 3D rendering.
(B) Dorsal view of the 3D rendering.
(C) Occipital (posterior) view of the 3D rendering.
(D) Coronal section (virtual), represented in dark grey in (A-C), selected to be just anterior to the level of the most posterior dorsal separation of the olfactory bulbs. Ethmoturbinate mean thickness and relative occupancy and skull roof compactness were measured in the ethmoid ROI and skull roof ROI, respectively.
Abbreviations: Soc, supraoccipital anteromedial; Nu, maximum thickness of the nuchal region; Par, parietal; Br, bregma; Fr, center of the frontal.

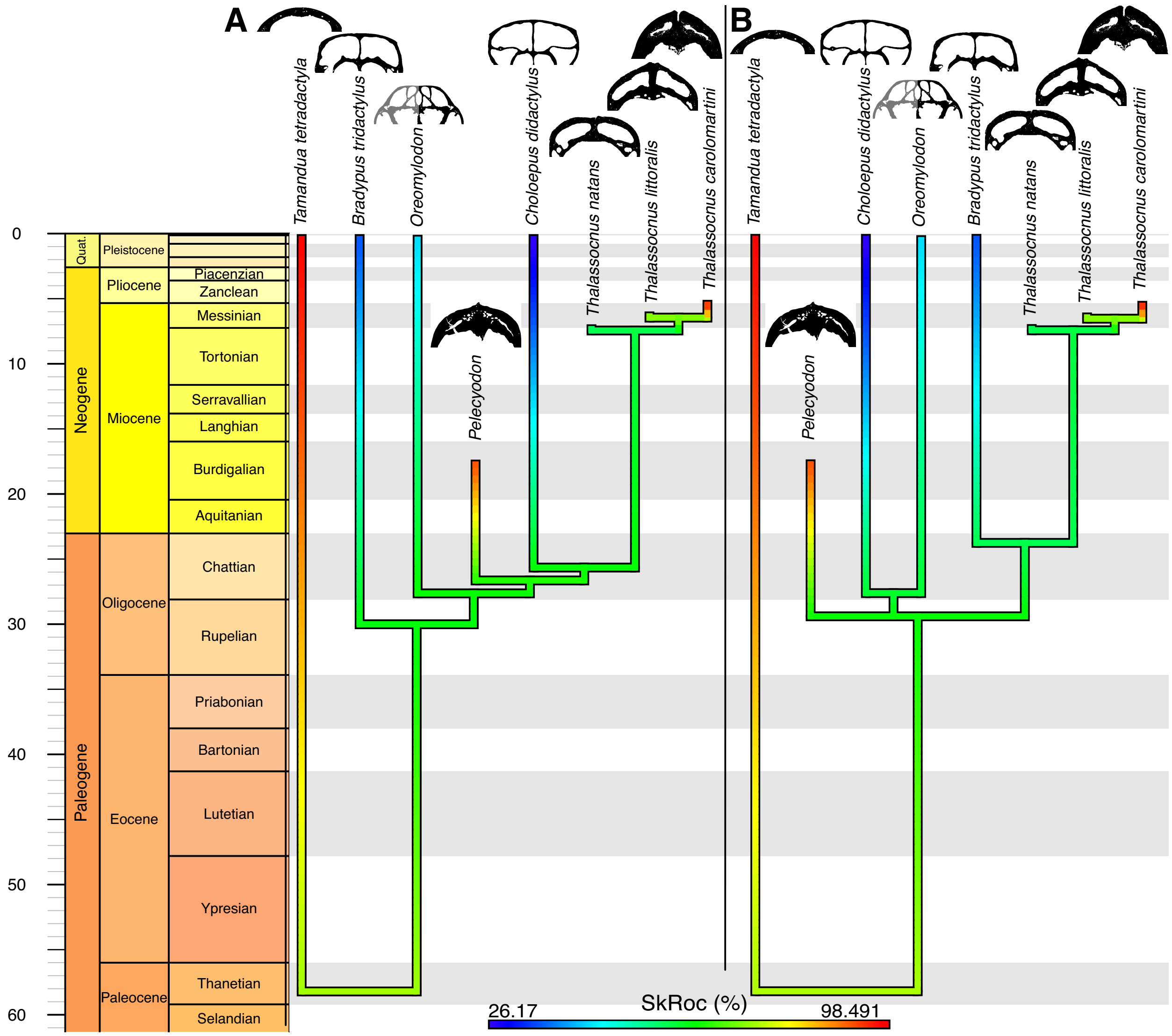


**Figure S2. Timetree with mapped skull roof compactness. Corresponding binarized skull vault sections are represented at the tips. Related to Figure 2.**

(A) ‘Morphological time-calibrated phylogeny’, based on [1–3] for extinct taxa topology and times of divergence, and [4] for the extant taxa times of divergence.

(B) ‘Mitochondrial time-calibrated phylogeny’, primarily based on [5], and on [1–3] for *Thalassocnus* interrelationship and ages of divergence (except time of divergence of anteaters and sloths, which is based on [4]).

The grey part of *Oreomylodon*’s section is reconstructed (not used for the measurement, which is made on one half of the skull in the case of the partial skull roof). Sections are not to scale.

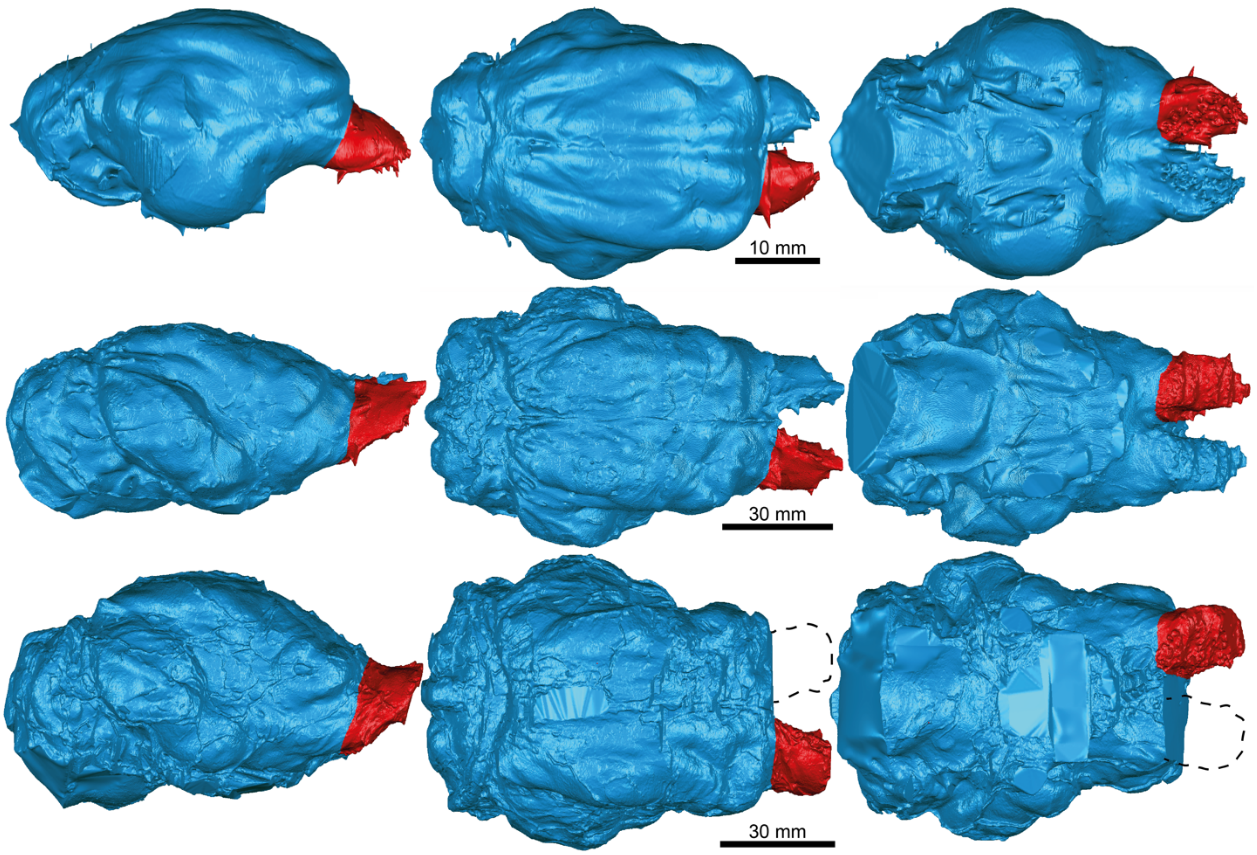


**Figure S3. Selection of the olfactory bulb for volume measurement in right lateral (left), dorsal (middle), and ventral (right) views. In red is selected the right bulb. Related to Figure 2.**

(A) *Bradypus tridactylus* (MNHN.ZM-MO1999-1065).

(B) *Thalassocnus natans* (MNHN.F.SAS734; postcranially non-pachyostotic and incipiently osteosclerotic).

(C) *Thalassocnus carolomartini* (SMNK-3814, mirrored; postcranially strongly pachyosteosclerotic).

## References


1. Amson E, Muizon C de, Gaudin TJ. 2016 A reappraisal of the phylogeny of the Megatheria (Mammalia: Tardigrada), with an emphasis on the relationships of the Thalassocninae, the marine sloths. *Zool. J. Linn. Soc.* (doi:10.1111/zoj.12450)
2. Muizon C de, McDonald HG, Salas R, Urbina M. 2004 The youngest species of the aquatic sloth *Thalassocnus* and a reassessment of the relationships of the nothrothere sloths (Mammalia: Xenarthra). *J. Vertebr. Paleontol.* **24**, 387–397. (doi:10.1671/2429a)
3. Ehret DJ, Macfadden BJ, Jones DS, DeVries TJ, Foster DA, Salas-Gismondi R. 2012 Origin of the white shark *Carcharodon* (Lamniformes: Lamnidae) based on recalibration of the Upper Neogene Pisco Formation of Peru. *Palaeontology* **55**, 1139–1153. (doi:10.1111/j.1475-4983.2012.01201.x)
4. Gibb GC, Condamine FL, Kuch M, Enk J, Moraes-Barros N, Superina M, Poinar HN, Delsuc F. 2016 Shotgun mitogenomics provides a reference phylogenetic framework and timescale for living xenarthrans. *Mol. Biol. Evol.* **33**, 621–642. (doi:10.1093/molbev/msv250)
5. Slater GJ, Cui P, Forasiepi AM, Lenz D, Tsangaras K, Voirin B, de Moraes-Barros N, MacPhee RDE, Greenwood AD. 2016 Evolutionary relationships among extinct and extant sloths: The evidence of mitogenomes and retroviruses. *Genome Biol. Evol.* **8**, 607–621. (doi:10.1093/gbe/evw023)

TO DELETE

**Figure 1. Evolution of cranial pachyosteosclerosis in the aquatic sloth *Thalassocnus*. Coronal sections (virtual) of the skull at the level of the posterior region of the olfactory bulbs (left) and just anterior to them (right).**
**(A) Extant sloth *Choloepus didactylus* (MNHN-ZM-MO-1996-594).**
**(B) *Thalassocnus natans* (MNHN-F-SAS-734; postcranially non-pachyostotic and incipiently osteosclerotic)**
**(C) *Thalassocnus littoralis* (MNHN-F-SAS-1615; postcranially of intermediate pachyosteosclerosis)**
**(D) *Thalassocnus carolomartini* (SMNK-3814; postcranially strongly pachyosteosclerosis)**
**Abbreviations: cp., cribriform plate; dnm., dorsal nasal meatus; et., ethmoturbinates; frs., frontal sinus; ns., nasal septum; ob., olfactory bulb endocast; pt., pterygoid. The scale does not apply to the smaller 3D rendering of the skull in (A). See also Movies** SErreur ! Nous n'avons pas trouvé la source du renvoi.-Erreur ! Nous n'avons pas trouvé la source du renvoi. **and Figure** SErreur ! Nous n'avons pas trouvé la source du renvoi..

**Figure 2. Quantification of the cranial pachyosteosclerosis and olfactory bulb volume ratio in the anteater and sloths. See also Table** SErreur ! Nous n'avons pas trouvé la source du renvoi. **and Figures S2-**Erreur ! Nous n'avons pas trouvé la source du renvoi.
**(A) Turbinate mean thickness (in mm), as measured in the ethmoid region of interest (ethmoid ROI; see Figure** SErreur ! Nous n'avons pas trouvé la source du renvoi.**D). Error bars indicate standard deviation. See also Figure 3.**
**(B) Turbinate compactness (in %). Proportion of the ethmoid ROI surface occupied by bone (see Figure** SErreur ! Nous n'avons pas trouvé la source du renvoi.**D).**
**(C) Skull roof compactness, as measured in the skull roof ROI (see Figure** SErreur ! Nous n'avons pas trouvé la source du renvoi.**D). The anteater *Tamandua* differs from sloths in the absence of frontal sinuses, biasing its value.**
**(D) Ratio of olfactory bulbs endocast to total brain endocast volume (%). See also Figure S3.**
**(E) Skull vault thickness at standard locations, as represented in a drawing in right lateral view of a skull of *Thalassocnus* (see also Figure** SErreur ! Nous n'avons pas trouvé la source du renvoi.**A-C). Locations with asterisk indicate no significant correlation to body size (as shown by linear regressions p-values > 0.05).**
**Location abbreviations: Soc, supraoccipital anteromedial; Nu, maximum thickness of the nuchal region; Par, parietal; Br, bregma; Fr, center of the frontal. Species abbreviations: B._tri, *Bradypus tridactylus*; C._di, *Choloepus didactylus*; Mega, *Megatherium*; Oreo, *Oreomylodon*; Pelec, *Pelecyodon*; Tam, *Tamandua*; T._caro, *T. carolomartini*; T._lit, *T. littoralis*; T._nat, *T. natans*.**

**Figure 3. Timetrees with mapped turbinate mean thickness. See also Table** SErreur ! Nous n'avons pas trouvé la source du renvoi. **and Figure** SErreur ! Nous n'avons pas trouvé la source du renvoi.
**(A) 'Morphological time-calibrated phylogeny', based on [1–3] for extinct taxa topology and times of divergence, and [4] for the extant taxa times of divergence.**
**(B) 'Mitochondrial time-calibrated phylogeny', primarily based on [5], and on [1–3] for *Thalassocnus* interrelationship and ages of divergence (except time of divergence of anteaters and sloths, which is based on [4]).**
