# Supplementary material for: Evolutionary adaptation to aquatic lifestyle in extinct sloths can lead to systemic alteration of bone structure
Source: Proc Biol Sci. 2018 May 9;285(1878):20180270. doi: 10.1098/rspb.2018.0270 (PMC5966604; doi:10.1098/rspb.2018.0270)
Supplement: Additional Results and Discussion [file rspb20180270supp4.pdf]

Evolutionary Adaptation to Aquatic Lifestyle in Extinct Sloths Can Lead to Systemic Alteration of Bone Structure

Eli Amson[1,2,3,*], Guillaume Billet[4], and Christian de Muizon[4].

[1]*Museum für Naturkunde, Leibniz-Institut für Evolutions- und Biodiversitätsforschung, Invalidenstraße 43; Berlin; 10115; Germany*
[2]*AG Morphologie und Formengeschichte, Institut für Biologie; Humboldt Universität zu Berlin, Philippstraße 13; Berlin; 10115; Germany*
[3]*Bild Wissen Gestaltung. Ein Interdisziplinäres Labor; Humboldt Universität zu Berlin, Sophienstraße 22a; Berlin; 10178; Germany*
[4]Centre de Recherche sur la Paléobiodiversité et les Paléoenvironnements-CR2P (CNRS, MNHN, UPMC, Sorbonne Universités), Département Origines et Évolution; Muséum national d'Histoire naturelle, 8 rue Buffon; Paris; 75005; France
[*]Correspondence to be sent to: *Humboldt Universität zu Berlin; Unter den Linden 6; 10099; Berlin; Germany. eli.amson@mfn.berlin*

Additional file 7: Supplementary Results and Discussion

Supplementary Results

Olfactory bulb endocast

Brain morphology was assessed based on segmented endocasts (electronic supplementary material, additional file 2 and file 3, Figure S3). Well-developed and individualized olfactory bulbs are found in all species of the aquatic sloth *Thalassocnus* (electronic supplementary material, additional file 3, Figure S3b-c, as in extant sloths ([1]; electronic supplementary material, additional file 3, Figure S3a), and terrestrial "ground sloths" [1,2]. The olfactory bulbs' endocast in the extant sloths *Bradypus* and *Choloepus* represent 2.9% and 4.1% of the total brain endocast, respectively (Figure 2d; electronic supplementary material, additional file 1). The ratios of all specimens of *Thalassocnus* fall between the two extant sloths' values. The olfactory bulbs of the late species of *Thalassocnus* are slightly smaller though, representing 3.7% in the early species against 3.0% in the latest species. Accordingly, the cribriform plate is also well-developed in all studied taxa.

Supplementary Discussion

Comparison with cetaceans

In extant cetaceans (which notably differ from *Thalassocnus* and sirenians by the fact that they are actively swimming predators), the olfactory system is reduced, involving a strong reduction/loss of the turbinates, reduction of the cribriform plate, and reduction/loss of the olfactory bulbs [3]. In the earliest members of the clade for which data are available (endocranial structure of earlier forms is poorly known), such as the "archaeocete" *Remingtonocetus* (middle Eocene), the skull is highly transformed with an elongate nasal cavity apparently mostly devoid of turbinates (at least anterior from the level of P4), even though the cribriform plate is present and nasal opening located at the tip of the snout [4] (some ethmoturbinates were present, directly anterior to the cribriform plate [5]). The middle Eocene protocetid *Aegyptocetus* [6] clearly shows few but complex and thin ethmoturbinates just anterior to the cribriform plate. The rest of the nasal cavity is left free of any turbinates. The skull walls are not conspicuously osteosclerotic (spongy inner structure at the external occipital protuberance for instance, see [6]: Supplementary Data 1). In the middle-late Eocene basilosaurid *Dorudon*, the cribriform plate is well developed, the ethmoturbinates are described as complex and thin, and other turbinates are not present (even where the ethmoturbinates are present they occupy only part of the nasal cavity; [7]). In the basilosaurid *Saghacetus*, nasoturbinates are described but are strongly reduced and simple [8]. On the whole, the earliest cetaceans for which we have data already show a strong reduction of the turbinates when compared to the general condition of terrestrial mammals. In an unidentified Eocene protocetid ([9]; and probably other taxa mentioned above), which is long-snouted, the olfactory bulbs were present at the tip of a long olfactory tract linking them to the rest of the brain, which indicate that, together with the presence of some remnants of turbinates (among other features), the olfactory morphology was comparable to that of the Recent balaenopterid mysticetes (the odontocetes featuring further reduction of the olfactory system [3]). The earliest cetaceans (some for which turbinate reduction is known) already show osteosclerotic postcrania (BMI is even found in the raoellid *Indohyus* [10]), of which the ribs, in some cases, are also pachyostotic [11–13].

REFERENCES

1. Gervais P. 1869 Mémoire sur les formes cérébrales propres aux édentés vivants et fossiles précédé de remarques sur quelques points de la structure anatomique de ces animaux et sur leur classification. *Nouv. Arch. du Musée d'Histoire Nat.* **5**, 1–56, 5 plates.
2. Dozo MT. 1987 The endocranial cast of an early Miocene edentate, *Hapalops indifferens* Ameghino (Mammalia, Edentata, Tardigrada, Megatheriidae). Comparative study with brains of recent sloths. *J. Hirnforsch.* **28**, 397–406.
3. Berta A, Ekdale EG, Cranford TW. 2014 Review of the cetacean nose: form, function, and evolution. *Anat. Rec.* **297**, 2205–2215. (doi:10.1002/ar.23034)
4. Bajpai S, Thewissen JGM, Conley RW. 2011 Cranial anatomy of middle Eocene *Remingtonocetus* (Cetacea, Mammalia) from Kutch, India. *J. Paleontol.* **85**, 703–718. (doi:10.1666/10-128.1)
5. Pihlström H. 2008 Comparative Anatomy and Physiology of Chemical Senses in Aquatic Mammals. In *Sensory Evolution on the Threshold Adaptations in Secondarily Aquatic Vertebrates* (eds JGM Thewissen, S Nummela), pp. 95–109. Berkeley: University of California Press. (doi:10.1525/california/9780520252783.003.0007)
6. Bianucci G, Gingerich PD. 2011 *Aegyptocetus tarfa*, n. gen. et sp. (Mammalia, Cetacea), from the middle Eocene of Egypt: clinorhynchy, olfaction, and hearing in a protocetid whale. *J. Vertebr. Paleontol.* **31**, 1173–1188. (doi:10.1080/02724634.2011.607985)
7. Uhen MD. 2004 Form, function, and anatomy of *Dorudon atrox* (Mammalia, Cetacea): an archaeocete from the middle to late Eocene of Egypt. *Univ. Michigan Pap. Paleontol.* **34**, 1–222.
8. Stromer E. 1903 *Zeuglodon*-reste aus dem Oberen Mitteleocän des Fajûm. *Beiträge zur Paläontologie und Geol. Österreich-Ungarns und des Orients* **15**, 65–100.
9. Godfrey SJ, Geisler JH, Fitzgerald EMG. 2013 On the olfactory anatomy in an archaic whale (Protocetidae, Cetacea) and the Minke whale *Balaenoptera acutorostrata* (Balaenopteridae, Cetacea). *Anat. Rec.* **296**, 257–272. (doi:10.1002/ar.22637)
10. Cooper LN, Thewissen JGM, Bajpai S, Tiwari BN. 2012 Postcranial morphology and locomotion of the Eocene raoellid *Indohyus* (Artiodactyla: Mammalia). *Hist. Biol.* **24**, 279–310. (doi:10.1080/08912963.2011.624184)
11. Houssaye A, Tafforeau P, Muizon C de, Gingerich PD. 2015 Transition of Eocene whales from land to sea: evidence from bone microstructure. *PLoS One* **10**, e0118409. (doi:10.1371/journal.pone.0118409)
12. Gray N-M, Kainec K, Madar SI, Tomko L, Wolfe S. 2007 Sink or swim? Bone density as a mechanism for buoyancy control in early cetaceans. *Anat. Rec.* **290**, 638–653. (doi:10.1002/ar.20533)
13. Madar SI. 1998 Structural adaptations of early archaeocete long bones. In *The emergence of whales, evolutionary patterns in the origin of Cetacea* (ed JGM Thewissen), pp. 353–375. New York: Plenum Press.
